# Prerequisites of sexual health literacy promoting service: a qualitative study in Iran

**DOI:** 10.1186/s12913-022-09018-7

**Published:** 2023-01-03

**Authors:** Raziyeh Maasoumi, Bita Jamali, Fatemeh Zarei, Mahmood Tavousi

**Affiliations:** 1 Department of Midwifery and Reproductive Health, School of Nursing and Midwifery, Tehran University of Medical Sciences, Tehran, Iran; 2 Nursing and Midwifery Research Care Center, School of Nursing and Midwifery, Tehran University of Medical Sciences, Tehran, Iran; 3 Department of Nursing and Midwifery, Comprehensive Health Research Center, Babol Branch, Islamic Azad University, Babol, Iran; 4 Department of Health Education and Health Promotion, School of Medical Sciences, Tarbiat Modares University, Tehran, Iran; 5 Health Metrics Research Center, Iranian Institute for Health Sciences Research, ACECR, Tehran, Iran

**Keywords:** Sexual health literacy, Sexual health services, Women of reproductive age, Iran

## Abstract

**Background:**

Sexual health literacy is one of the determinants of the rate, factors, and outcomes of problems associated with sexual health and reproductive. The prerequisite of having and promoting this type of literacy is the appropriate organization and access to sexual health services. The current study was conducted with the objective of describing the perceptions and experiences of health care providers and recipients of sexual health literacy promoting services.

**Methods:**

The current qualitative study was conducted on 37 individuals in the form of 3 individual in-depth and semi-structured interviews and 4 focus group discussions (26 individuals) with service recipients (women of reproductive age), and 8 in-depth and semi-structured interviews with service providers in Amol from September 2019 to March 2020. The recorded interviews were transcribed and analyzed through content analysis.

**Results:**

Data analysis resulted in the extraction of the theme titled “prerequisites of sexual health literacy promoting service” which consisted of two subthemes ‘client-oriented service’ and ‘efficient service’. In the client-oriented service attention must be paid to the client’s age, sex, needs and socio-cultural background. Efficient service is a service which is based on continuing sexual education, can reconstruct sexual attitudes, is professional, team-based, integrated into other services, and has a follow-up structure.

**Conclusions:**

The results outline the requirements for sexual health literacy promoting services which should be taken into consideration during the policymaking, planning and design of relevant health programs.

## Background

Sexual health literacy (SHL) is a set of knowledge, beliefs, attitudes, incentives, and personal abilities in the access, perception, assessment, and utilization of sexual health information in one’s daily life, which enables and empowers a person to judge and decide to create change in one’s sexual life [1]. Based on the World Health Organization’s (WHO) report in 2016, SHL will play a significant role in achieving some of the Millennium Development Goals, including ending poverty and famine, equal education, gender equality and empowerment of women and girls, economic growth, and reduction of inequality within and between countries [2]. The promotion of SHL –particularly in women- can be an effective and efficient strategy for reducing burden of sexual health diseases by helping women to building knowledge about sexual health risks and agency in sexual wellbeing and subsequently reduce unwanted pregnancies and sexually transmitted diseases and promote sexual interactions between couples [3, 4]. In fact, SHL is one of the determinants of the rate, factors, and outcomes of sexual health related problems and reproductive.

Based on various studies, multiple factors affect sexual health, including age, gender, place of birth, education, and sexual experience [5], stigma, relationships, HPV vaccination status, current structural factors [1] –such as religious attachments [5], cultural, social and economic factors, social support of public service providers [1], and the sources of receiving reproductive and sexual health services [6]. It may thus be said that SHL is the skill and ability to utilize health information –which is influenced by a set of contextual and historical factors, and various health services and emerging technologies [7].

In their study, Tuteja et al. (2017) report that access to education is one of the determinants of reproductive and sexual health literacy. These researchers reported verbal education along with active participation and involvement of clients in discussions as the most effective strategies of promoting reproductive and SHL [8]. Martin et al. (2017) also recommended a wide range of purposeful interventions to increase SHL with a focus on stigma, positive messages, and development of interactive and critical skills. They also emphasize that the role of contextual and key factors such as official health services and access to correct online and offline information were effective in the shaping of SHL [1]. Vongxay et al. (2019) also reported raising awareness and skill strengthening as strong predicting variables of reproductive and sexual health literacy [9]. Therefore, it seems that the organization and access to appropriate and relevant sexual health services is one of the main determining factors in the provision and promotion of SHL [10]. Moreover, the dynamic interaction between sexual health service provider and recipient can also lead to the acquisition of SHL [7]. The SHL acquired in this interaction can lead to the adoption of sexual and reproductive behavior such as reduction of high-risk sexual behavior, STDs, unwanted pregnancies, and play a significant role in the shaping of a safe sexual life [11–13].

### Sexual health literacy and relevant service deliveries: Iran’s status

Most SHL studies in the world have been conducted on high-risk groups such as adolescents and youth, students, immigrants, and male homosexuals [1, 5, 7, 9]. While, in developing countries such as Iran the study of SHL among all age and gender groups including women of reproductive age is important. Currently, more than 22 million people of the entire population of the country are women of reproductive age [14]. Women of reproductive age are among the main reproductive foundations of the population. Furthermore, women have an important role in safeguarding, providing, and promoting the family’s health given their maternal and spousal roles in the family structure [15]. The SHL status of women of reproductive age in Iran is not exactly clear. Jamali et al’s study (2020) examined SHL among women of reproductive age in one of Iran’s northern cities and showed that 23.3% of the target audience had limited SHL [16]. The study’s researchers believed that these results may somehow have been overestimated and this may be due to the error of overestimating women’s sexual knowledge and their low perceived threat of these problems and sex-related risks in the population under study.

In Iran, there are services wherein efforts are made to raise service recipients’ SHL. Educational classes have been offered to couples at the time of marriage in Iran since 1993 by MOHME (Ministry of Health and Medical Education) in the country’s healthcare centers. These classes have been able to provide couples with the minimum required levels of reproductive and sexual education. Further sexual health services provided by Iran’s primary healthcare (PHC) services include education and assessment of the status of STDs, screening of common cancers, and sexual problems. These services begin with the service providers’ (particularly midwives) pre-determined set of questions outlined in the IHS (the Integrated Health System). In case there are problems that need therapeutic interventions, the clinically provided services are relatively good. However, in case the clients do seek special and sexual health therapeutic services, there is no specifically outlined service process or referral in the system for such needs. Therefore, the access and quality of sexual health services received have been one of the main challenges of delivering such services in Iran’s PHC system [17, 18]. Given sexual health services are one the significant factors of providing and promoting SHL, and that SHL is a concept that is affected by contextual factors, society’s socio-cultural structure, and the target audiences’ traits [1, 5, 19], thus, the current study was conducted to describe the perceptions and experiences of health care providers and recipients for establishing the sexual health literacy services in health care services in future.

## Methods

The current study is a qualitative study that has employed the conventional content analysis approach in Amol (one of Iran’s northern cities) during September 2019 and March 2020 [20]. The research setting includes four selected healthcare centers across the city and 5 private gynecology and psychology clinics in Amol. The research population included women of reproductive age receiving healthcare and therapeutic services from midwives, gynecologists, reproductive specialists and psychologists –as providers of relevant services. Purposeful sampling was done with maximum variety until saturation was reached. Data were collected through in-depth and semi-structured interviews and FGDs by BJ as the corresponding author. The purpose of the FGD is to compare different opinions and collect data in a short period of time to discover important information about the target group [21]. This method is appropriate for studying sensitive topics such as topics related to sexual health [22].

At first, with the permission of the health centers, many women’s mobile numbers were taken from the Integrated Health System, then some of them agreed to participate in the interviews after calling and explaining the objectives of the research. Then time and place of the interviews were announced to them as service recipients.

Midwives, gynecologists, reproductive specialists and psychologists who had experience, skills and expertise in the field of sexual and reproductive health as well as be Willing to participate in the study were recruit in the part of data collection related to health care service providers. They were interviewed at their location of service or private clinics, with prior appointments, their informed consent, and safeguarding their privacy. First, 3 individual semi-structured interviews were conducted with women of reproductive age. Then, 4 FGDs (a total of 26 individuals) were conducted with women of reproductive age receiving services in which there were 6 people in three groups and 8 people in one group; two of these discussions were held with academically educated women and the other two were held with women with non-academic degrees, bearing in mind maximum age variation. Moreover, 8 individual interviews were held with service providers including 3 midwives, 1 reproductive health specialist, 2 clinical psychologists, and 2 gynecologists.

The selection criteria of the recipients of the service were, age: 18–49, being originally Iranian, being literate, having been married, not having any current disease under treatment or special diseases, being an urban citizen, not being pregnant, not being in the puerperium period, not having academic degrees in medical and paramedical sciences, not being a healthcare personnel, and willing to participate in the study. The service providers were selected from those who had professional experience in providing sexual health services. The service recipients were selected from a maximum variation of age, socio-economic and educational levels (Table 1).

**Table 1:** Demographic characteristics of service recipients

Characteristics		(***n*** = 28)
**Age**	< 30 years	4 (11.92%)
	30–39 years	12 (44.04%)
	40–49 years	12 (44.04%)
**Years of marriage**	0–10	9 (32.14%)
	11–21	12 (42.85%)
	22–32	7 (25.01%)
**Number of children**	0–1	14 (50%)
	2–3	14 (50%)
**Educational status**	non-academic degrees	14 (50%)
	academic education	14 (50%)
**Employment status**	Housewife	20 (71.43%)
	Employed	8 (28.57%)

The interviews were semi-structured and to collect data in the service recipients ‘section (both in interviews and FGD), BJ as the second author started with a general question “What do you do to obtain the sexual information you need?” and then continued with questions such as, “How would you verify the authenticity of this information?”. Furthermore, she asked service recipients to describe their perceptions and experiences on the services provided to them to raise their SHL and further continued by asking exploratory questions such as, “Has attending the center and receiving services been helpful?”; “If yes, what characteristic did the service you received have that fulfilled your need?”; “If not, which characteristics should your required service have?”. During data collection, service providers were asked to describe their perception and professional experience in providing and promoting SHL among their clients by asking questions such as “What do you do to provide your clients’ sexual health?” and “Which information do you give them?”. Thereafter, BJ continued the interview with exploratory questions like “Have the services offered by you been able to provide your clients with sexual health literacy, skills and applied knowledge?” If yes, “What characteristics did your services have?” “If not, what characteristics should these services have?”. The interviews were 45 to 70 minutes long.

### Data analysis

We analyzed the data using the conventional content analysis approach and pattern recommended by Erlingsson & Brysiewicz (2017) [20]. Based on this pattern, after every interview the corresponding author (BJ) transcribed and typed the interviews in the first possible opportunity.

The semantic units or in other words, the collection of words that carried a specific meaning or concept with regards to the research goal were summarized and similar primary codes were placed in one subcategory. Afterwards, the subcategories were reviewed many times and based on their similarities and differences with each other were placed into a category; similar categories formed the main theme [22]. The textual data and extracted codes were managed using MAXQDA 2018 software.

### Trustworthiness

To evaluate and increase the scientific rigor of the findings we used the method recommended by Lincoln and Gabba. The credibility of data was ensured by prolonged engagement with the data, data immersion, performance of in-depth interviews in multiple sessions, and member checking. To allow member checking, The interviews were implemented in a word file after execution. 5 of these files were given to 5 interviewees to assess the accuracy of the transcriptions. Confirmability was done with external check peer debriefing and by the research team and by two faculty members specialized in qualitative research to examine the documented interviews as well as the codes and categories extracted.

The dependability of the data was checked by three reproductive and sexual health specialists outside the research team who were skilled in qualitative research, to have their supervision over the data analysis process and to make use of their confirmatory or critical suggestions. Confirmability was done by accurately registering the path of the research and decisions adopted herein and documenting them. For transferability purposes the service recipients were selected upon considering maximum variability in age, education, and socio-economic status [23].

### Ethical approval

This study is part of a combined study of a PhD dissertation on reproductive health which has been approved by the Ethics Committee of Tehran university of Medical Sciences under ethics code IR.TUMS.FNM.REC.1398.077. Formal licenses were acquired before entering the research setting. All participants were aware of the research goal and had the right to leave the study whenever they wished. Moreover, we asked the participants for permission to audio-record the interviews. They were assured that all findings would remain confidential. Eventually, all those who agreed to participate in the interviews signed the informed consent form.

## Results

The service-receiving participants’ age ranged from 25 to 49 years and their mean age was 32.9 years. The service providers’ age ranged from 31 to 51 years and their mean age was 41 years. Further details of the participants’ demographic characteristics (both service providers and service recipients) are presented in Tables 1 and 2.

**Table 2:** Demographic characteristics of interviewed service providers

Number	Healthcare Provider	Age	Workplace	Professional experience (Years)
1	Midwife 1 (Bachelor)	51	Health center	16
2	Midwife 2 (Bachelor)	49	Health center	21
3	Midwife 3 (Bachelor)	48	Health center	24
4	Gynecologist	49	Private clinic	11
5	Gynecologist	41	Private clinic	4
6	Psychologist (PhD)	31	Private clinic	6
7	Psychologist (PhD)	46	Private clinic	15
8	Reproductive health specialist(PhD)	46	Private clinic	18

**Fig. 1:**
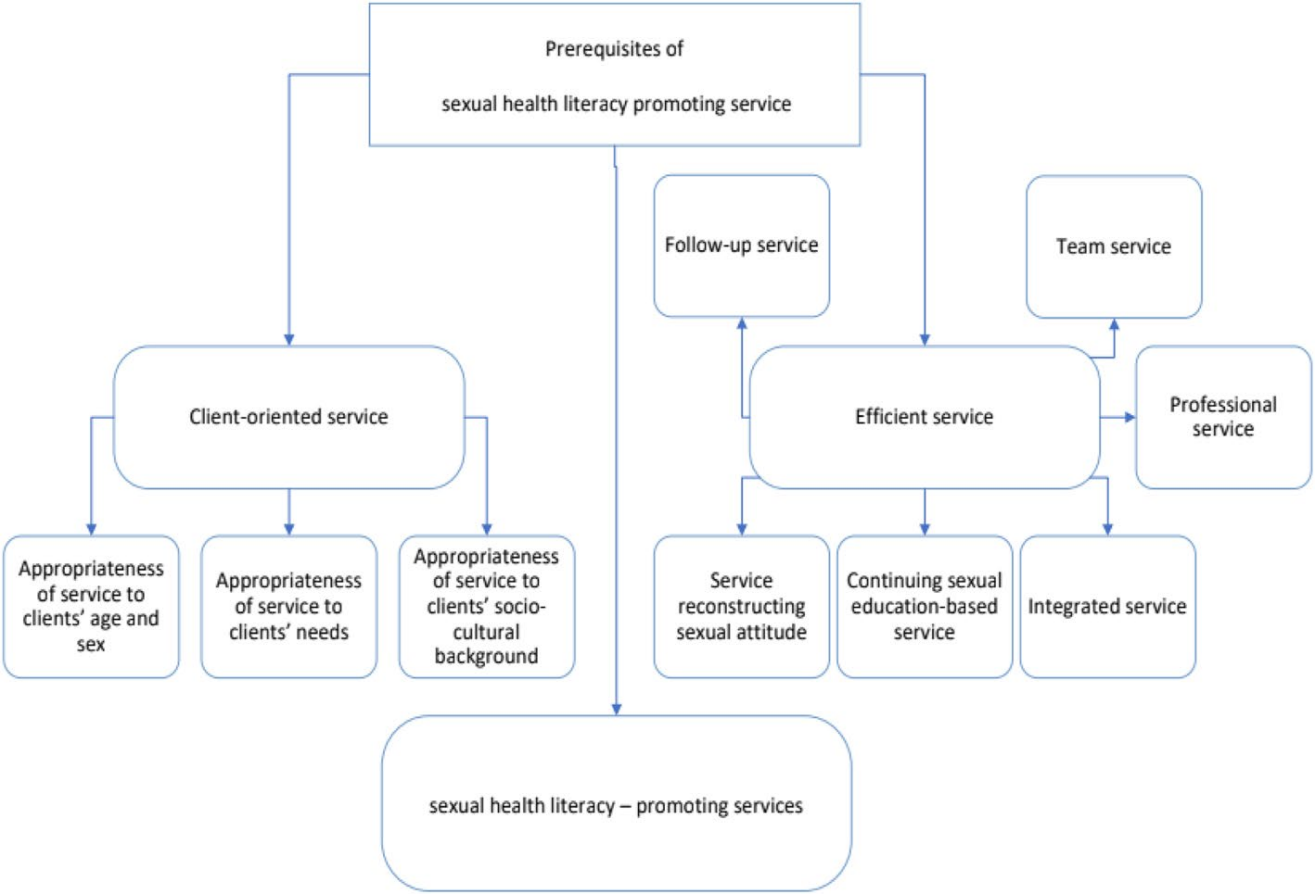
Services promoting sexual health liter

Data analysis resulted in one theme titled ‘prerequisites of sexual health literacy promoting services, which further consisted of two main categories titled ‘client-oriented service’ and ‘efficient service’ and 9 subcategories. The categories, subcategories, and code examples are presented in Table 3 and the overall schema of sexual health literacy promoting service is illustrated in Fig. 1.

**Table 3:** Needs and perceptions of health care providers and service recipients on the prerequisites of sexual health literacy promoting services

Categories	Subcategories	Code examples
Client-oriented service	Appropriateness of service to clients’ needs	• The need for counseling on sexual issues • Requesting information to have safe sex [2] • Provision of information through health care centers bearing in mind each age group’s needs
	Appropriateness of service to clients’ age and sex	•The necessity of sexual health education based on individuals age and sex • Sexually educating men appropriate to their age
	Appropriateness of service to clients’ sociocultural background	• The impact of religious factors on dealing with sexual issues • Notification and education of sexual matters to obviate unnecessary shame and modesty
Efficient service	Continuing sexual education-based service	• The need for continuous sexual education delivered by service providers • The need for continuous education of sexual information to all individuals of the society
	Integrated service	• Integration of sexual health education into other health services • Teaching sexual health to individuals simultaneously alongside other health services
	Professional service	• Specialized education of sexual health to service providers • Service providers in the sexual health domain should possess adequate knowledge and communication skills • Correcting the belief that reception of sexual information is obscene
	Service reconstructing sexual attitude	• Correcting public attitudes and misconceptions about sexual matters
	Team service	• Time-saving and clients convenience in working with teams • The necessity of teamwork between the midwife and psychologist in centers to solve clients’ problems
	Follow-up service	• Service delivery to clients based on follow-up • Using text messaging to send reminders and following up clients

### Client-oriented service

Client-oriented service was identified as the first category of SHL promoting services. This category addresses the conditions or characteristics which are appropriate to the clients’ circumstances based on the service which is intended to promote SHL. The subcategories described in this category include appropriateness of service to clients’ age and sex, appropriateness of service to clients’ needs and appropriateness of service to clients’ socio-cultural background.

#### Appropriateness of service to clients’ age and sex

All participants (both service providers and service recipients) believed that the services should be suitable to individuals age and sex, as each person has one’s own specific issues and problems at a specific age, thus requiring information and services suitable to one’s own conditions.

*“At the beginning of my marriage when I was younger, I really needed sex related information. I still do. I have a couple of questions about sex related issues for which I don’t know where to look for the right answers or who to talk to. I think these services should be provided to men too. My husband needs to acquire information and services appropriate to his age as well”. (37-year-old service recipient, high school graduate, married for 13 years).*

Also, many participants complained about their lack of information in the field of sexual health in menopause and wanted training from service providers. They believed that sexual health was forgotten in this era.

*I am very confused about what will happen to my sexual life after menopause and I don’t know where to get information about this age” (service recipient, married for 21 years).*

#### Appropriateness of service to clients’ needs

To promote their SHL the service recipients recommended training and counseling appropriate to each age group separately.

Many of the service recipients and the service providers placed emphasis on receiving information, education, and counseling on learning about the opposite sex and how to deal with one’s spouse, the quality of sex, sexual intercourse and becoming pregnant, unusual sexual relations and their complications, the vast variety of sexual issues and appropriately dealing with them.

*“We must teach our women about quality sex, factors leading to sexual problems, sexual abuse behaviors and how to protect their sexual health. Based on my experience, many of the women do not have correct information about the items I just referred to and proceed with their sex lives on a trial-and-error basis, while, if they did have the necessary skills and information, they would have much better sex and marital lives” (service provider, 16 years of professional experience in health care centers).*

#### Appropriateness of service to clients’ socio-cultural background

Both service providers and service recipients believed that the information, training, and services that are provided in the field of sexual health should be appropriate to the society’s sociocultural structure.

They believed that sexual health planning and education should be done based on the cultural and religious conditions of the people; Eliminating and breaking taboos in educational programs, making new and full content of educations available in the media and better introducing the realities of the society through the media in accordance with the existing socio-cultural conditions of the society, can play a positive role in promoting sexual health literacy.

*“At the beginning of my marriage, I wouldn’t be sexually satisfied and I had difficulties with my spouse. After much searching, I consulted a counselor. S/he told me to masturbate; I was very upset by her suggestion and scorned myself for going to such a person in the first place! I kept thinking to myself “is this the only solution to this problem?! Must I commit a sin to enjoy my lawful relationship with my husband?!” Isn’t masturbation forbidden in our religion?” (28-year-old service recipient, bachelor’s degree holder, married for eight years).*

*“I’ve seen many of my clients bear unnecessary shame and modesty; for example, I had a young female client who had a very good emotional relationship with her husband but unfortunately, she was so shy in her sexual relationship and had so much unnecessary shame and modesty that her husband had begun to complain. Modesty is one of our societies cultural, religious, and value concepts. However, this very modesty sometimes ruins things. Modesty has no place in sex, neither in terms of religion nor custom. Such matters should be taught to couples so that they can have more quality sexual lives” (service provider, psychologist, 15 years of professional experience).*

### Efficient service

This category describes the characteristics of the SHL promoting service at structural level. In other words, the characteristics of an efficient service in the PHC system to promote SHL has been described. The subcategories included in this category are: continuing sexual education-based service, service reconstructing sexual attitude, professional service, integrated service, team service, and follow-up service.

#### Continuing sexual education-based service

In this study, the service providers laid emphasis on receiving continuing sexual education. In their belief, sexual training must be continually delivered by service providers.

*“Having information is always good, particularly about these issues where we don’t receive proper training from anywhere. Training must be continually delivered to people by centers and individuals whose profession and specialty this is” (40-year-old service receiver, master’s degree holder, married for 18 years).*

Service providers believed that continuing sexual education was a necessity for operationalizing SHL programs and that teaching the primary principles of health and describing the concept of sexual health to people were the first, most important, and simplest ways to promote SHL. They thought that continuing sexual education was necessary and that people needed to acquire continuing information, skills, and applied knowledge in the field of sexual health so that their sexual health would be provided for.

*“All people need to learn about sexual issues… so we should have continuing sexual education. People’s communications should not be limited to the service delivery system; they should be continually taught. I always try to keep this in mind during my work, but there are very few people who think like me. Furthermore, our working circumstances sometimes don’t give us the liberty to act otherwise. We should have a structure for continuing sexual education in the system” (service provider, gynecologist, 4 years professional experience).*

#### Service reconstructing sexual attitude

All participants (both service providers and service recipients) believed that to promote SHL, negative attitudes towards sexual topics should be eliminated and the vulgarity of receiving sexual information should be corrected/modified. Therefore, by creating positive attitudes and destroying taboos inappropriate behaviors that have become commonplace in the society through incorrect information or beliefs should be modified.

*“I think to raise sexual literacy and information among the society the belief that this is inappropriate and bad should be eliminated. We should be able to express our questions, concerns and issues easily and personnel should be able to guide us correctly and give us information” (36-year-old service recipient, high school graduate, married for 14 years).*

*“As long as sexual issues are a taboo in society nothing can be done. First, people’s attitudes and beliefs towards sexual issues should be corrected and then sexual health literacy can be expected to improve” (service provider, gynecologist, 11 years of professional experience).*

#### Professional service

Service providers stated that to enter the sexual health field –be it giving information, education, or counseling to clients to raise their SHL-, they should have received professional training in this field beforehand. Many of them believed that during their education they had not acquired the specialized knowledge and skills, thus lacking the interest and incentive to enter the field. Having basic counseling skills such as effective communication and the principles of performing correct counseling, possessing advanced skills such as specialized knowledge in the field of sexual health and professional therapeutic and counseling skills in this field were further mentioned by them as being absent.

*“Personally, I don’t have much expertise in sexual health knowledge, as we were not taught these topics during our education. It’s very important that we are taught the necessary skills in this field. If a service is to be provided, I think one of the most important issues is to teach these skills to service providers or those who are currently being trained in line with their discipline and specialty” (service provider, gynecologist, 4 years of professional experience).*

#### Team service

Service providers believed that their teamwork would lead to the proper identification and resolution of clients’ problems through team collaboration and discussion.

*“In the center, we are a team, working side by side with the psychologist. Whenever we need other specialties, we refer the clients. Those who are outside our setting are familiar with our work routine and cooperate with us. I think that the clients receive services very well in this manner. Their time is saved and their job is done by a coordinated team” (service provider, midwife, 24 years of professional experience in a private center).*

#### Integrated service

In the current study, all participants thought that it is better that information skills and applied knowledge in the field of sexual health are integrated into other health care services. That is, sexual health services should be integrated into primary health care along with other services in order to promote sexual health literacy.

*“It would be very good if I am provided with correct information in this field and I am told what to do and what not to do whenever I go to a health center” (27-year- old service recipient, high school graduate, married for six years).*

*“Services should be provided in integrated form. For example, when a pregnant woman comes for her prenatal visits it’s better that she receives sexual health education while she is receiving prenatal care” (service provider, midwife, 21 years of professional experience in health care centers).*

#### Follow-up service

Service delivery is valuable and effective if associated with the client’s follow-up. Service providers believed that many services are delivered in the health system but there is no robust guideline or supervisory system for following up the clients to assess the effectiveness of the services. They also added that in many cases they are unaware of their client’s aftermath once the service is delivered. In their opinion, if the service is to be provided to promote SHL in the system it must be followed up.

*“I always emphasize to my client that she should follow-up her treatment. I think the same rules should apply to sexual issues. Clients should show follow-up their therapies. The service provided in this field should be designed on a follow-up basis” (service provider, midwife, 21 years of professional experience in health care centers”.*

Furthermore, service providers stated that some service recipients do not follow-up due to negligence or their preoccupations. Therefore, they believed it would be suitable to follow-up clients through telephone contact and/or text messages.

*“We take the individuals’ cellphone numbers and the secretary reminds them through a text message. This is how we follow-up our clients” (service provider, reproductive specialist, 18 years of professional experience).*

## Discussion

The current study’s data analysis yielded the ‘prerequisites of sexual health literacy promoting service’ theme, which further consisted of two categories, ‘client-oriented service’ and ‘efficient service’. Client-oriented service is a service in which the client’s age, sex, needs, and sociocultural background should be kept in mind. Efficient service is a service which is based on continuing sexual education, can reconstruct sexual attitudes, is professional, team-based, integrated into other services, and has a follow-up structure.

By ‘appropriateness of service to clients’ age and sex’ we mean access to sexual health services and education appropriate to their age and sex. Bostani et al. (2020), who also conducted a study aimed at describing sexual health education strategies based on empowerment, observed that teaching sexual health and modifying the educational structure to empower individuals should be done appropriate to the target group’s characteristics. It is necessary to provide sexual health education that is appropriate to individuals’ age and sex and based on their information needs and skills, to empower them toward safeguarding and promoting their sexual health [24, 25].

In explaining the ‘appropriateness of service to the clients’ needs’ we may say that the need to provide and promote information, skills, and applied knowledge –particularly to improve the quality of women’s sexual relations- was one of the most prominent points raised in the current study. In Khani et al’s study (2018), which was conducted to examine the most common needs of sexual and repropductive health in Sari, the results indicated that women in all fields of reproductive and sexual health needed to receive information, thus, provision of services that can lead to the provision of such needs and promote SHL seems necessary [26]. In Bostani et al’s study, the results indicated that sexual health education should be based on the needs of the target group. Therefore, needs assessment and the involvement of the target group appear to be necessary for promoting sexual health too [24].

Analysis of the ‘appropriateness of service to the clients’ sociocultural background’ indicated that all participants believed that those services can promote SHL which are within the framework of sociocultural considerations and religious values [25]. Svensson et al. (2017) conducted a study aimed at exposing immigrant women to culturally sensitive sexual health information and examining its impact on their health literacy. Based on their findings, women’s perceptions of sexual and repropductive health issues were deeply affected by cultural norms, gender structures, pudency, and their internal taboos [27]. According to Bostani et al’s study sexual health education must be based on the target group’s culture and the most effective way to ensure success of health interventions is to identify the current structures among the target group and act according to their cultural sensitivities [24].

Continuing sexual health education includes the services of sexual health which are continuous and persistent; the persistence of this service is the requisite of functionalizing SHL programs. McDaid et al. conducted a study to determine the conceptual framework of SHL among homosexual men and learned that the continual teaching of appropriate health information to individuals is crucial to promoting their SHL [20]. Sexual health education planners can assess and teach sexual health in the form of short-term and long-term continuing education courses, by training experts in this field –even if the issue is needed by clients but not expressed by them [24]. This way, sexual health literacy may be promoted.

In the subcategory of services reconstructing sexual attitudes, all participants believed that culture building and modification of sexual attitudes should be done to be able to promote people’s sexual health level [24]. Simpson (2015) believes that belief or attitude structures play important roles in SHL, such that a person’s attitude can provide the framework for the effectiveness of different sexual health interventions [5]. Bostani et al. (2017) also acknowledged that improving one’s attitude toward sexual issues is the prerequisite of one’s empowerment in sex [28], both of which are consistent with our study’s results.

With respect to professional service, it was concluded that health personnel -themselves- should have adequate knowledge in the field of sexual health. Moreover, access to quality sexual care will be ensured through health care providers proper skills and by ensuring client privacy [29]. Moghaddam et al. (2018) listed health personnel problems such as lack of their (physician and midwife) familiarity with clients’ sexual needs and their unaccountability and the absence of a trained counselor in health centers as the causes of insufficiency of health care centers in delivering sexual health services [30], findings which are consistent with ours.

McDaid et al. (2021) showed that service providers’ scientific and communication skills are central and important in promoting SHL. To achieve this, service providers should have effective communication skills and adequate information regarding sexual health concerns among different groups of the society. Furthermore, they should be well prepared to respond to the target groups’ questions [7].

Service providers believed that teamwork with other specialists at the site of service would familiarize them with the clients’ problems and resolve them. Rozeboom et al. (2012) also indicated that team members from different scientific disciplines should collaborate with each other to achieve the desirable outcome [31].

In the integrated service subcategory, all participants believed that to meet the sexual health demands of individuals, the health service system should create certain changes and the delivery of sexual health services should be integrated into the repropductive health service system [32]. Mogahaddam et al. (2018) identified integration of services in proper coordination with the referral system and social services as a desirable strategy for providing special health needs, a finding which was confirmed by our results. The integration of sexual health education in PHC is very important to allow individuals greater control over their sexual health through greater awareness and complete and continuous access to information in the field of sexual health [33]; a point that should be taken into consideration by policymakers.

In the follow-up service subcategory, we found that service delivery alone was not sufficient and that service delivery should be associated with follow-up for its effectiveness. Jessup et al. (2018) designed an intervention to raise health literacy in a hospital population; the service providers believed that it is better if they receive complete information for follow-up along with a letter of instructions before being discharged and that physicians communicate well with them so they can ask their questions. Moreover, if a person does not attend for follow-up, they should be informed through a phone call and that the follow-up visit should be long enough to allow for their queries [34].

The reasons stated by the clients for not following up their problems in Moghaddam’s study were: fear of diagnostic and therapeutic methods, spontaneous improvement and economic poverty, insensitivity to exposure to high-risk behaviors– which can itself indicate the client’s unawareness, the significance of education and awareness, and the necessity of its continuation by service providers for an effective follow-up [30]. In our study too the clients believed increased awareness as effective factors for following up their problems.

Subsequently, by linking education to follow-up of sexual and reproductive health services, and holding courses on continuing sexual education, counseling and communication skills for service providers, sexual health services will also be promoted.

Among the limitations of the current study was the sensitivity of the subject which affected the participants incentives and inclination to enter the study; which also relatively prolonged the sampling duration. By describing the goals of the study and building trust the researcher attempted to recruit participants into the study and to manage this limitation.

## Conclusion

Based on the results, a client-oriented service is a service in which the client’s age, sex, need, and sociocultural background must be taken into consideration. An efficient service is a service which is based on continuing sexual education, can reconstruct sexual attitudes, is professional, team-based, integrated into other services, and has a follow-up structure. These two important principles are the prerequisites of designing SHL promoting services, which should be considered by health care planners, managers, and policymakers.

## Data Availability

The data that support the findings of this study are available from the corresponding author [BJ] upon reasonable request.
